# ﻿*Paphiopedilummotuoense* (Orchidaceae, Cypripedioideae), a new species from Motuo, Xizang, China

**DOI:** 10.3897/phytokeys.259.145861

**Published:** 2025-06-30

**Authors:** Feng-Xia Tang, Wen-Hui Rao, Ze Zhang, Xin-Yi Wu, Meng Wang, Jian Li, Jie-Shan Chen, Xiao-Juan Duan, Mei-Na Wang

**Affiliations:** 1 Shenzhen Key Laboratory for Orchid Conservation and Utilization, Key Laboratory of National Forestry and Grassland Administration for Orchid Conservation and Utilization, The Orchid Conservation & Research Center of Shenzhen and the National Orchid Conservation Center of China, Shenzhen, 518114, Guangdong, China The Orchid Conservation & Research Center of Shenzhen and the National Orchid Conservation Center of China Shenzhen China; 2 Yunnan Yelantang Biotechnology Co., Ltd, Kunming, 650000, Yunnan, China Yunnan Yelantang Biotechnology Co., Ltd Kunming China

**Keywords:** China, new orchid species, *Paphiopedilummotuoense*, plastid genome

## Abstract

*Paphiopedilummotuoense*, a newly discovered orchid from Motuo County, Xizang, China, is described and illustrated. *Paphiopedilummotuoense* can be distinguished from the related *Paphiopedilum* species *P.qingyongii* and *P.venustum* by several distinctive features: a significantly smaller staminode above the column, a distinct purplish-red lip with purplish-brown veins, pouched with erect and triangular auriculas on both sides of its mouth, and leaves with pale green and pale yellowish-green tessellations. The novelty is also well supported as a new species by molecular phylogenetic analysis. Additionally, the complete chloroplast genome of *P.motuoense*, 157,218 bp in length, was assembled and annotated. It contains an LSC region of 86,275 bp, SSC region of 949 bp and two IRs of 34,997 bp, with 120 genes, including 76 PCGs, 36 tRNA genes and 8 rRNA genes.

## ﻿Introduction

Orchidaceae, one of the two largest angiosperm families with more than 29,000 species, is divided into five subfamilies (Chase et al. 2015; Govaerts et al. 2021; Zhang et al. 2023; Pérez-Escobar et al. 2024). The subfamily Cypripedioideae Lindl. (slipper orchids) is characterized by a pouchlike lip, two fertile stamens, a shield-like staminode, and a synsepal composed of the fused lateral sepals (Lindley 1840; Dressler 1982; Guo et al. 2012). Slipper orchids have a wide distribution, ranging from temperate to tropical regions of Eurasia and America, and the conduplicate-leaved species native to tropical Asia were considered a distinct lineage, which was established the genus *Paphiopedilum* by Ernst Hugo Heinrich Pfitzer in 1886 (Pfitzer 1886; Cox et al. 1997; Guo et al. 2012).

*Paphiopedilum* is the largest genus of the subfamily Cypripedioideae, comprising 109 accepted species and 35 natural hybrids (Liu et al. 2009; IPNI 2023; POWO 2024). Based on morphological characteristics, Pfitzer (1903) summarized the taxonomic studies of *Paphiopedilum* and divided the genus into three subgenera, namely *Anotopedilum*, *Brachypetalum* and *Otopedilum*. Brieger (1971) maintained the delimitation of *Brachypetalum* subgenus, and divided the genus into four subgenera (*Barbata*, *Brachypetalum*, *Paphiopedilum* and *Polyantha*). Later, Karasawa and Saito (1982) combined the data of chromosome number, divided the genus into six subgenera (*Brachypetalum*, *Cochlopetalum*, *Paphiopedilum*, *Parvisepalum*, *Polyantha* and *Sigmatipetalum*), and all the sections of Barbata subgenus were put into the Sigmatipetalum subgenus; but this delimitation result was not widely accepted. Atwood (1984) merged the delimitation of Karasawa and Saito (1982), and divided the genus into only two subgenera (*Brachypetalum* and *Paphiopedilum*) based on the lip morphology. Then, Cox et al. (1997) performed molecular phylogenetic analyses based on nuclear rDNA ITS sequences and Cribb (1997), based on morphological characteristics of flower inflorescence, leaf type, floral morphology and molecular phylogenetic results, and divided the genus into three subgenera (*Brachypetalum*, *Paphiopedilum* and *Parvisepalum*). The subgenus Paphiopedilum, characterized by having a helmet-shaped or slipper-shaped pouch, a rather long claw at the base, and lacking the incurved or involute margins at the apex, comprises 93 accepted species and is divided into five sections (*Barbata*, *Cochlopetalum*, *Coryopedilum*, *Paphiopedilum* and *Pardalopetalum*) (Cribb 1997; Tsai et al. 2020; Guo et al. 2021; POWO 2024). Widespread reticulate evolution within *Paphiopedilum*, coupled with the discovery of new species like *P.canhii* (Averyanov 2010) and *P.rungsuriyanum* (Gruss 2014), results in intricate interspecific relationships. Additional data and further research are needed to understand these relationships (Guo et al. 2012, 2021; Tsai et al. 2020).

*Paphiopedilum* is mainly distributed in tropical and subtropical southeast Asia (Cribb 1987; Cribb 1997; Liu et al. 2009; Pemberton 2013). With 33 accepted species, China is an important species diversity center of *Paphiopedilum* (Liu et al. 2009; Liu et al. 2020; POWO 2024; COL-China 2024; IPNI 2025). In China, *Paphiopedilum* species are predominantly distributed across the southwestern to southern regions, with the highest species diversity recorded in Yunnan Province (Liu et al. 2009; POWO 2024; COL-China 2024). Recent taxonomic studies have identified several novel species within Yunnan Province, such as *P.notatisepalum* Z.J.Liu, Meina Wang & S.R.Lan (Wang et al. 2017). Xizang, constituting the principal region of the Qinghai-Tibet Plateau, harbors the world's highest-latitude tropical rainforest and represents a plant diversity hotspot with the greatest elevational range globally. To date, only five *Paphiopedilum* species have been documented in Xizang (Chen et al. 2023), including *P.fairrieanum* (Lindl.) Stein (Stein 1892), *P.micranthum* Tang & F.T.Wang (Tang and Wang 1951), *P.qingyongii* Z.J.Liu & L.J.Chen (Liu and Chen 2010), *P.venustum* (Wall. ex Sims) Pfitzer (Pfitzer 1888) and *P.wardii* Summerh. (Summerhayes 1932).

*Paphiopedilum* species are extremely rare and highly prized for their ornamental value, notably their exceptionally attractive flowers (Cribb 1987; Cribb 1997; Liu et al. 2009; Pemberton 2013). All *Paphiopedilum* species have been included in Appendix I of the Convention on International Trade in Endangered Species of Wild Fauna and Flora (CITES), the specimens of which are prohibited in international trade except for non-commercial purposes (https://cites.org/eng). In China, all *Paphiopedilum* species have also been listed on the List of Wild Plants under State Priority Conservation, and their illegal collections have been forbidden since 2021.

During a botanical investigation in Motuo County, Xizang, China, in 2014, we found a distinct subpopulation of *Paphiopedilum* under the broad-leaved forest. Some of these plants were transferred to the Yunnan Yelantang Biotechnology Co., Ltd. (Kunming, China) at that time. Based on the monitoring of those plants and the detailed morphological and molecular comparisons with similar species, they were considered to belong to a new species of *Paphiopedilum* which is described below.

## ﻿Materials and methods

### ﻿Morphological analysis

Мorphological characteristics of the new species, including the number, length and width of leaf, the length and width of scape, the diameter and color of flower, the shape, length and width of sepal, petal, and staminode, were observed, measured and photographed based on three flowering living individuals of this species in Motuo, Xizang, China. The terminologies we used follow the terms of Chen et al. (2009) and Liu et al. (2009). The voucher specimen is deposited in the National Orchid Conservation Center of China (NOCC). We compared its morphological characteristics with 87 species descriptions in the literature of *Paphiopedilum* genus, and with 9 herbarium specimens belonging to *P.qingyongii* (including holotype) and *P.venustum*. These herbarium specimens of the two species are housed in NOCC, National Plant Specimen Resource Bank Main Library (PE), Herbarium of Kunming Institute of Botany, Chinese Academy of Sciences (KUN), and Tibetan Plateau Biological Herbarium of the Chinese Academy of Sciences (HNWP).

### ﻿Molecular analysis

Total genomic DNA was extracted from fresh leaf (voucher specimen J.B.Chen 01800) using a modified procedure of CTAB (cetyltrimethylammonium bromide) (Doyle and Doyle 1987; Li et al. 2013). After purifying the extracted DNA, we prepared paired-end libraries by fragmenting the genomic DNA into short fragments of approximately 350 bp. Subsequently, the libraries were sequenced in paired-end (150 bp) using the Illumina NovaSeq 6000 platform (Illumina, Inc., San Diego, CA, USA) at Novogene Co., Ltd. (Beijing, China). A total of 10 GB reads of genome skimming data were generated.

The plastid genome was assembled employing GetOrganelle with appropriate parameters (Jin et al. 2020), using the chloroplast genome of *P.venustum* (Wall. ex Sims) Pfitze as the reference sequence (OM066335). The Bandage software was employed to visualize the final assembly graphs of the plastid genomes, assessing their completeness and accuracy (Wick et al. 2015). Then, the obtained scaffolds and contigs were annotated by Geneious Prime (Biomatters Ltd., Auckland, New Zealand) (Kearse et al. 2012) and Plastid Genome Annotator (Qu et al. 2019). The annotated complete chloroplast genome was deposited in GenBank with accession number OR392426. The circular plastid genome map of *P.motuoense* was drawn and visualized in Chloroplot online software (https://irscope.shinyapps.io/chloroplot/) (Zheng et al. 2020). A total of 62 species from *Paphiopedilum* were included in the molecular phylogenetic analyses, with *Phragmipediumlongifolium* (Warsz. & Rchb.f.) Rolft (NC_028149) used as the outgroup (Li et al. 2016; Guo et al. 2021) (Table 1). We used the Python script get_annotated_regions_from_gb.py (https://github.com/Kinggerm/PersonalUtilities) to extract protein-coding gene sequences from the plastid genome. Each protein-coding gene was individually aligned by MAFFT 7.3 (ffT-NS-i × 1000 strategy) (Katoh and Standley 2013) and removed by trimAl 1.2 with default settings (Capella-Gutiérrez et al. 2009). Finally, the 67 protein-coding genes were rapidly concatenated using the Python script concatenate_fasta.py (https://github.com/Kinggerm/PersonalUtilities) before phylogenetic analyses.

**Table 1.** Morphological comparisons of *P.motuoense* and its two related species.

Characters	*Paphiopedilummotuoense*	*P.venustum*	*P.qingyongii*
Leaf pattern and color	leaves adaxially tessellated with **pale green and pale yellowish green**	leaves adaxially tessellated with dark green and gray- or yellow-brownish green	leaves adaxially without tessellation or scarcely tessellated
Petal color	**petals pale yellowish white**, flushed with purplish red	petals yellow-whitish with green veins, flushed with dark red in apical half	petals downward curvy, white-green densely spotted with purple-red
Lip color	**lip purplish red with purplish brown veins**, pouch margin pale yellowish white	lip pale yellow, distinctly veined with green and slightly flushed dark red, pouch margin pale yellow	lip pale yellow, distinctly veined with deep green, pouch margin yellow
Pouch mouth of lip	**pouch triangular auriculate on both sides of its mouth**	pouch auriculate on both sides of its mouth	pouch auriculate on both sides of its mouth
Staminode shape and size	staminode **smaller**, reniform-obcordate, **2.9–3.2 × 5–5.3 mm**	staminode larger, reniform-obcordate, 6–7 × 9–13 mm	staminode larger, broadly oblong, 6.9–7.1 × 8.8–9 mm

Phylogenetic analyses were performed using Maximum Likelihood (ML) and Bayesian inference (BI). The ML analysis was conducted using IQ-TREE v1.6 with SH-aLRT test and ultrafast bootstrap (UFBoot) feature (–alrt 1000 –bb 1000 –nt AUTO) (Nguyen et al. 2015; Hoang et al. 2018). The substitution model for the concatenated alignment was automatically selected using ModelFinder in IQ-TREE, with the Bayesian Information Criterion (BIC) identifying TVM+F+R2 as the best-fit model. BI analyses of phylogeny were performed in MrBayes v.3.2.6 under partition model (2 parallel runs, 2000000 generations), in which the initial 25% of sampled data were discarded as burn-in. The produced phylogenetic trees were rendered using Figtree v1.4.4 (http://tree.bio.ed.ac.uk/software/figtree/) and enhanced with Adobe Illustrator software for improved visualization.

## ﻿Results and discussion

### ﻿Morphological evidence

**Figure 1.**
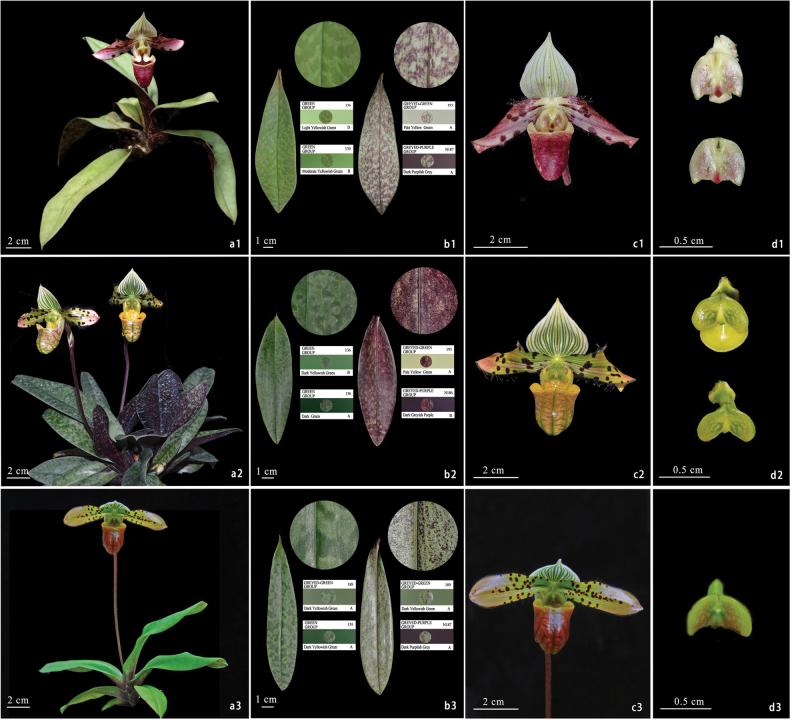
*Paphiopedilummotuoense* and its allies **a1–d1***P.motuoense***a1** whole plant **b1** leaf **c1** flower **d1** staminode. **a2–d2***P.venustum***a2** whole plant **b2** leaf **c2** flower **d2** staminode. **a3–d3***P.qingyongii***a3** whole plant **b3** leaf **c3** flower **d3** staminode.

The leaf morphology, petal and lip color, pouch mouth trait, and staminode size that the novelty shows are distinguishing characteristics of *Paphiopedilum*, which indicates the novelty is placed within the genus. Moreover, the novelty exhibits the closest similarities with *P.venustum* and *P.qingyongii*, but differs in several key characteristics, including triangular auriculas on both sides of its pouch mouth, a significantly smaller staminode above the column, a distinct purplish-red lip with purplish-brown veins, pale yellow-white petals flushed with purple-red spots, and leaves tessellated with pale green and pale yellowish-green spots. These compared morphological characters of the novelty, *P.venustum*, and *P.qingyongii*, are clearly presented in Table 1 and Fig. 1, according to which we speculate that this novelty could be a new species, and we name it *P.motuoense*.

### ﻿Molecular evidence

**Figure 2.**
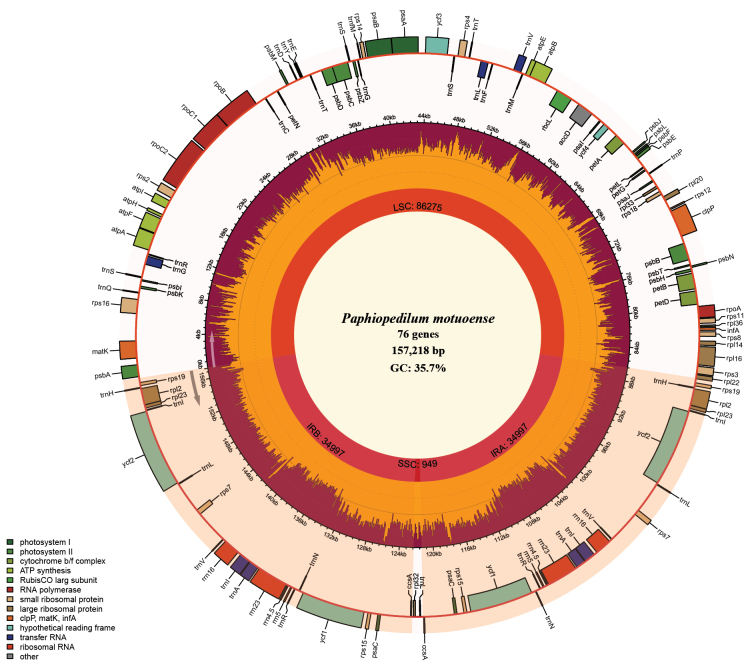
Chloroplast genome map of *Paphiopedilummotuoense*.

**Figure 3.**
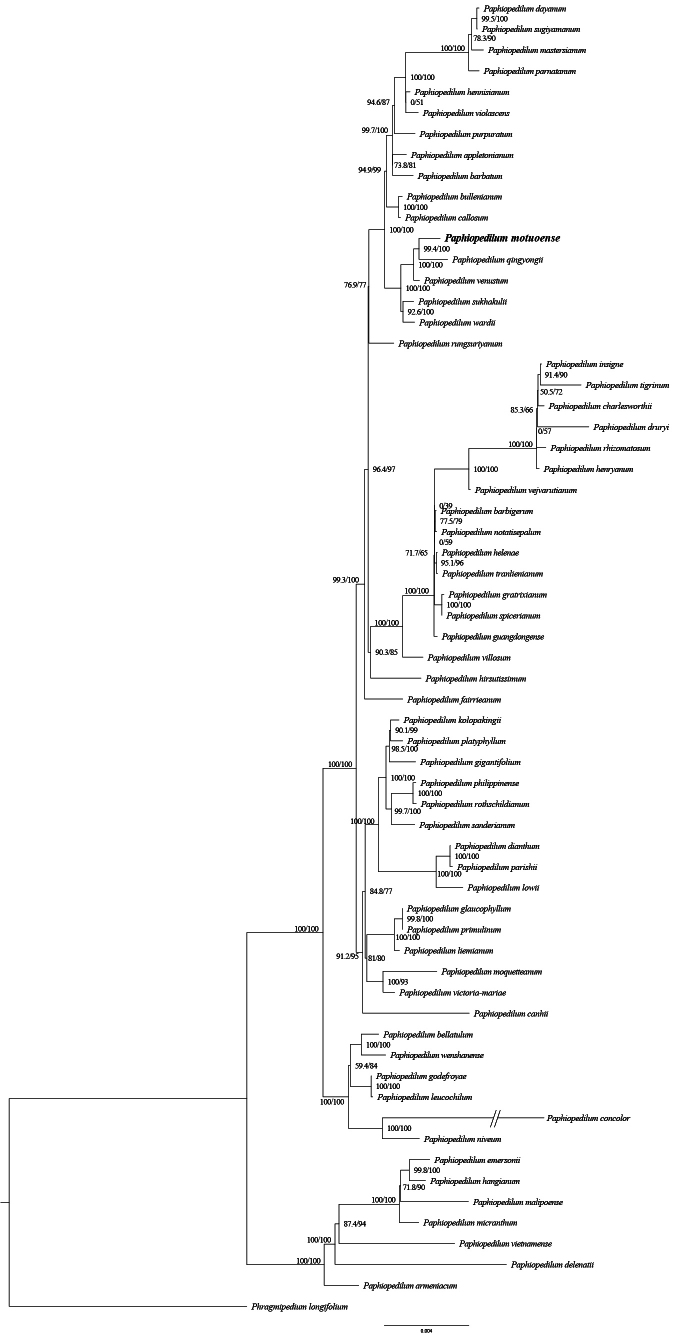
Phylogram of *Paphiopedilum* spp., based on 67 shared protein-coding genes from 62 *Paphiopedilum* whole plastomes. The numbers near the nodes are the values of SH-aLRT test (left) and the ultrafast bootstrap (right).

The complete plastid genome of *P.motuoense* is 157,218 bp in length and exhibits a typical quadripartite structure, including one large single-copy region (LSC) of 86,275 bp, one small single-copy region (SSC) of 949 bp, and two inverted repeat regions (IRs) of 34,997 bp (Fig. 2). The chloroplast genome contains 120 genes, including 76 protein-coding genes (PCGs), 36 tRNA genes and 8 rRNA genes (Table 2, Fig. 2). The total GC content of the whole plastome is 35.7%. The phylogenetic analyses based on 67 shared protein-coding genes from 62 *Paphiopedilum* whole plastomes indicate that *P.motuoense* is close to *P.qingyongii* with high support (SH-aLRT 99.4%, UfBoot 100%) and then sister to *P.venustum* also with strong support (SH-aLRT 100%, UfBoot 100%) (Fig. 3). The BI tree (Suppl. material 1) exhibits identical topological relationships to the ML analysis.

**Table 2.** Genes presented in the chloroplast genome of *P.motuoense*.

Group of genes	Gene
Photosystem I	*psaA*, *psaB*, psaC*, *psaI*, *psaJ*
Photosystem II	*psbA*, *psbB*, *psbC*, *psbD*, *psbE*, *psbF*, *psbH*, *psbI*, *psbJ*, *psbK*, *psbL*, *psbM*, *psbN*, *psbT*, *psbZ*
Cytochrome b/f complex	*petA*, *petB*, *petD*, *petG*, *petL*, *petN*
ATP synthase	*atpA*, *atpB*, *atpE*, *atpF*, *atpH*, *atpI*
NADH dehydrogenase	–
Rubis CO large subunit gene	*rbcL*
RNA polymerase	*rpoA*, *rpoB*, *rpoC1*, *rpoC2*
Small ribosomal proteins	*rps2*, *rps3*, *rps4*, rps7*, *rps8*, *rps11*, *rps12*, *rps14*, rps15*, *rps16*, *rps18*, rps19*
Large ribosomal proteins	rpl2*, *rpl14*, *rpl16*, *rpl20*, *rpl22*, rpl23*, *rpl32*, *rpl33*, *rpl36*
rRNA	rrn4.5*, rrn5*, rrn16*, rrn23*
tRNA	trnA-UGC*, *trnC-GCA*, *trnD-GUC*, *trnE-UUC*, *trnF-GAA*, *trnfM-CAU*, *trnG-GCC*, *trnG-UCC*, trnH-GUG*, trnI-CAU*, trnI-GAU*, trnL-CAA*, *trnL-UAA*, *trnL-UAG*, *trnM-CAU*, trnN-GUU*, *trnP-UGG*, *trnQ-UUG*, trnR-ACG*, *trnR-UCU*, *trnS-GCU*, *trnS-GGA*, *trnS-UGA*, *trnT-GGU*, *trnT-UGU*, trnV-GAC*, *trnV-UAC*, *trnY-GUA*
Translational initiation factor	*infA*
Maturase	*matK*
C-type cytochrome synthesis	ccsA*
Subunits of Acetyl-CoA-carboxylase	*accD*
Protease	*clpP*
Conserved open reading frames	ycf1*, ycf2*, *ycf3*, *ycf4*

Note: *means duplicated gene in IRs.

Our morphological and molecular evidences indicate that *P.motuoense* is distinctive for identification as a separate species from the other *Paphiopedilum* species. The following is a description of the *P.motuoense*.

### ﻿Taxonomy treatment

#### ﻿ Paphiopedilum motuoense

Taxon classificationPlantaeAsparagalesOrchidaceae

M. N. Wang, F. X. Tang & W. H. Rao sp. nov.

3CFD05AF-7E92-5659-9E38-9CD52D31C602

urn:lsid:ipni.org:names:77364576-1

**Figure 4.**
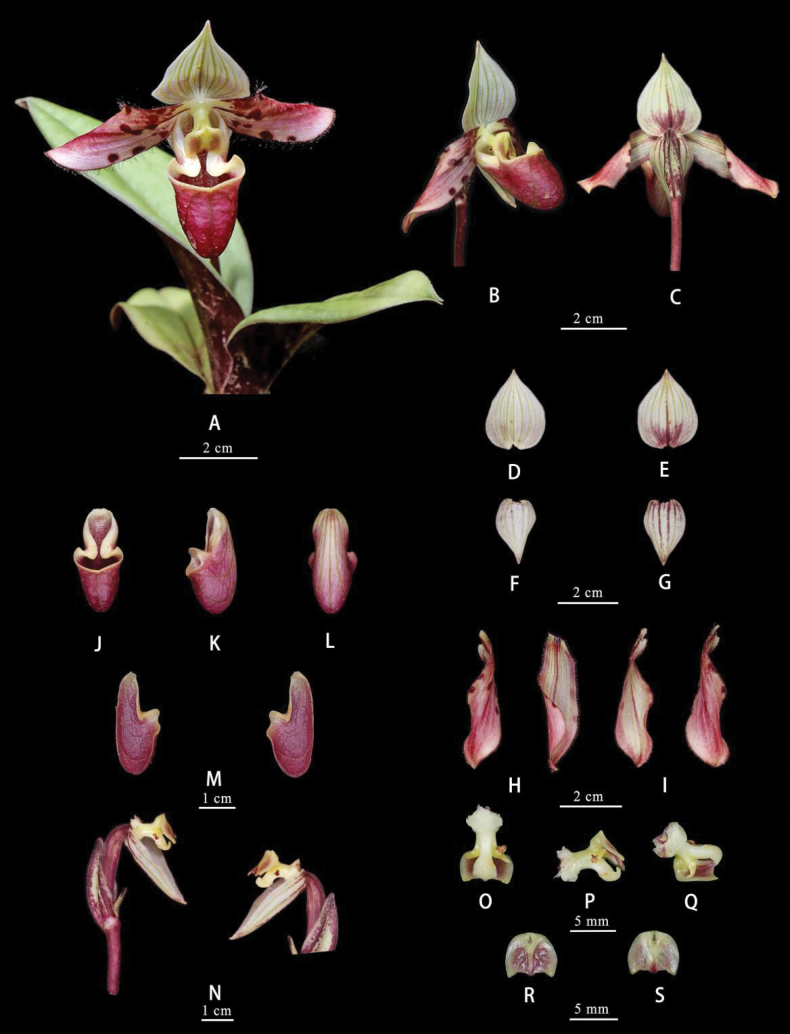
Images of living plants of *Paphiopedilummotuoense***A** whole plant **B, C** flower (side view and back view) **D, E** dorsal sepal (front view and back view) **F, G** synsepal (front view and back view) **H, I** petal (front view and back view) **J–L** lip (front view, side view and back view) **M** lip (vertical section) **N** ovary and column (with bract, synsepal and staminode) **O–Q** column (back view and side view) **R, S** staminode (front view and back view).

**Figure 5.**
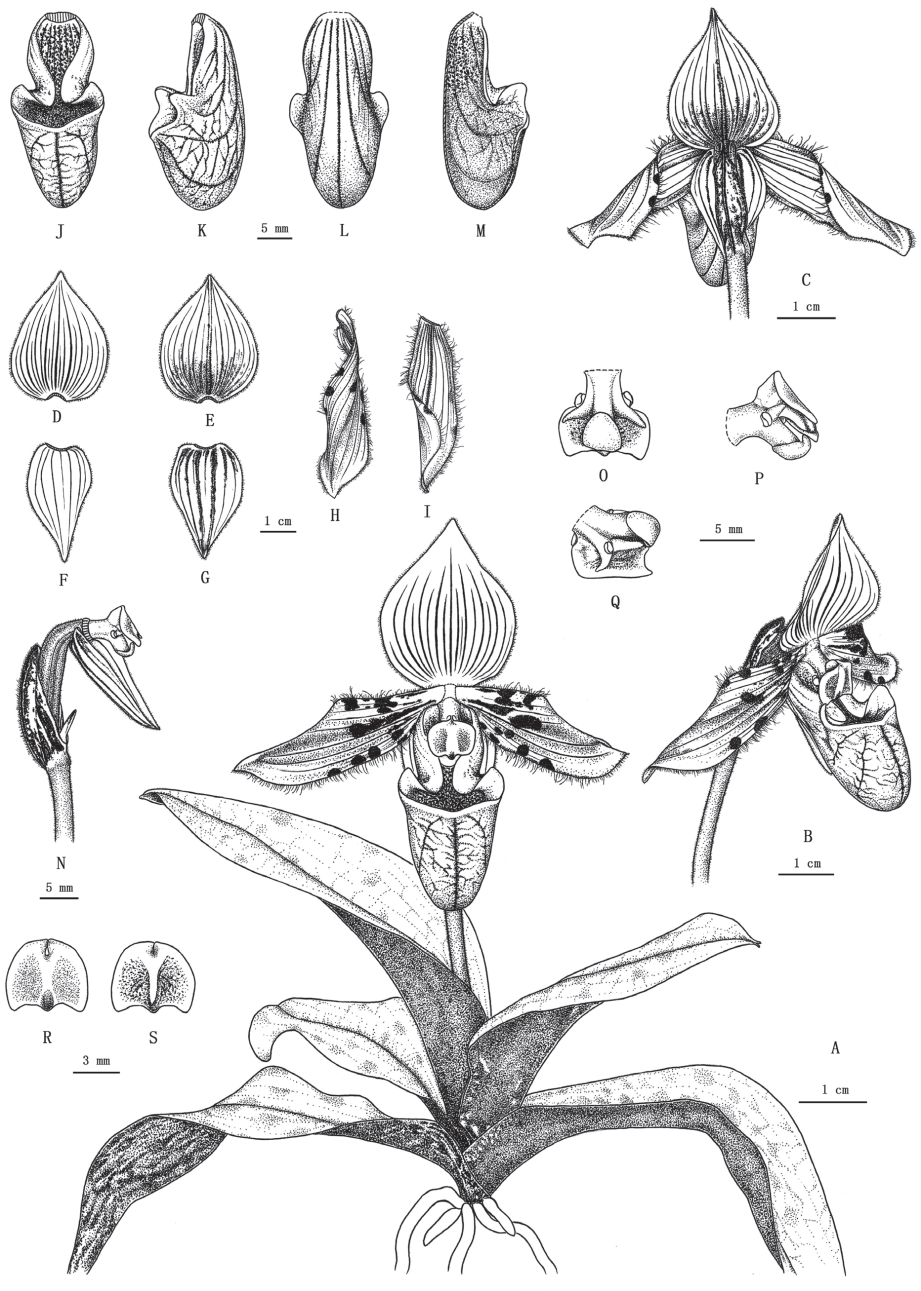
*Paphiopedilummotuoense***A** whole plant **B, C** flower (side view and back view) **D, E** dorsal sepal (front view and back view) **F, G** synsepal (front view and back view) **H, I** petal (front view and back view) **J–L** lip (front view, side view and back view) **M** lip (vertical section) **N** ovary and column (with bract, synsepal and staminode) **O–Q** column (back view and side view) **R, S** staminode (front view and back view).

Figs 4 , 5 Chinese name. 墨脱兜兰

##### Type.

China • Xizang, Linzhi City, Motuo County, alt. 800 m, 23 Apr 2021, *J.B.Chen 01800* (holotype: NOCC).

##### Diagnosis.

*Paphiopedilummotuoense* is similar to *P.venustum* and *P.qingyongii* in morphology, but differs from them by having a smaller staminode, petals flushed with purplish red, a purplish red lip with purplish brown veins, pouched with erect and triangular auriculas on both sides of mouth, and leaves adaxially tessellated with pale green and pale yellowish green (Table 1, Fig. 1).

##### Description.

Plants terrestrial. Leaves 5–6, basal, oblong, 5–8 × 1.6–1.9 cm, apex acute and slightly 2-lobed, adaxially tessellated with pale green and pale yellowish green, abaxially densely spotted with purple, base contracted into petiole shape. Scape erect, up to 5.3–7.1 cm long, purple, densely shortly hirsute, 1-flowered, terminal; bract broadly ovate, 2.1–2.3 × 1.6–1.8 cm, margin ciliate; pedicel and ovary 2.6–3.1 cm long, pubescent. Flower 5.7–6.2 cm in diam.; dorsal sepal and synsepal white with green veins; petals pale yellowish white, flushed with purplish red, with a few large maroon warts mainly in basal half; lip purplish red with purplish brown veins, margin pale yellowish white; staminode pale yellow with green veins and flushed with pale purplish red. Dorsal sepal obcordate, 3.2–3.5 × 2.6–2.7 cm, apex acuminate, abaxially pubescent, margin ciliate; synsepal ovate, 3.2–3.4 × 1.8–2 cm, apex acute, abaxially pubescent, margin ciliate. Petals obovate-oblong, 4.4–4.7 × 1.6–1.8 cm, apex obtuse or subacute, margins long-ciliate; lip helmet-shaped, 2.4–2.7 cm long; lateral lobes without verruca or slightly verrucose; pouch 1.4–1.6 × 1–1.1 cm, slightly narrowed toward base, with triangular auriculas on both sides of its mouth, margins erect at mouth apex, villose at inner bottom, minutely papillate-puberulent outside. Staminode reniform-obcordate, 2.9–3.2 × 5–5.3 mm, with a sinus at apex, sinus with mucro at middle, adaxially finely papillate.

##### Etymology.

The species epithet “*motuoense*” refers to the locality name of type specimen of this new species, Motuo County, Linzhi City, Xizang, China.

##### Distribution and habitat.

*Paphiopedilummotuoense* is currently known only from the type locality Motuo County, Xizang, China. It is terrestrial in humus-rich places and semi-epiphytic in the humus of rocky places in forest margins at elevations of 800 m.

##### Phenology.

Flowering in March and April.

## Supplementary Material

XML Treatment for Paphiopedilum motuoense

## ﻿Additional information

### Author ORCIDs

Feng-Xia Tang https://orcid.org/0000-0003-2575-9665

Wen-Hui Rao https://orcid.org/0000-0002-2177-6700

Ze Zhang https://orcid.org/0000-0002-8774-4867

Xin-Yi Wu https://orcid.org/0000-0002-8623-1867

Meng Wang https://orcid.org/0000-0002-1665-3642

Jian Li https://orcid.org/0000-0002-0096-6257

Jie-Shan Chen https://orcid.org/0009-0000-8859-6053

Xiao-Juan Duan https://orcid.org/0009-0000-0429-8594

Mei-Na Wang https://orcid.org/0000-0001-5998-6028

### Data availability

All of the data that support the findings of this study are available in the main text.

## Supplementary materials

Supplementary material 1 Bayesian Inference (BI) phylogenetic tree of *Paphiopedilum* species Data type pdf Explanation note Based on chloroplast genome sequences. https://binary.pensoft.net/file/1364182This dataset is made available under the Open Database License (http://opendatacommons.org/licenses/odbl/1.0/). The Open Database License (ODbL) is a license agreement intended to allow users to freely share, modify, and use this Dataset while maintaining this same freedom for others, provided that the original source and author(s) are credited.Feng-Xia Tang, Wen-Hui Rao, Ze Zhang, Xin-Yi Wu, Meng Wang, Jian Li, Jie-Shan Chen, Xiao-Juan Duan, Mei-Na Wang
