# Supplementary figures and images for: ﻿Paphiopedilummotuoense (Orchidaceae, Cypripedioideae), a new species from Motuo, Xizang, China
Source: PhytoKeys. 2025 Jun 30;259:131–44. doi: 10.3897/phytokeys.259.145861 (PMC12238957; doi:10.3897/phytokeys.259.145861)

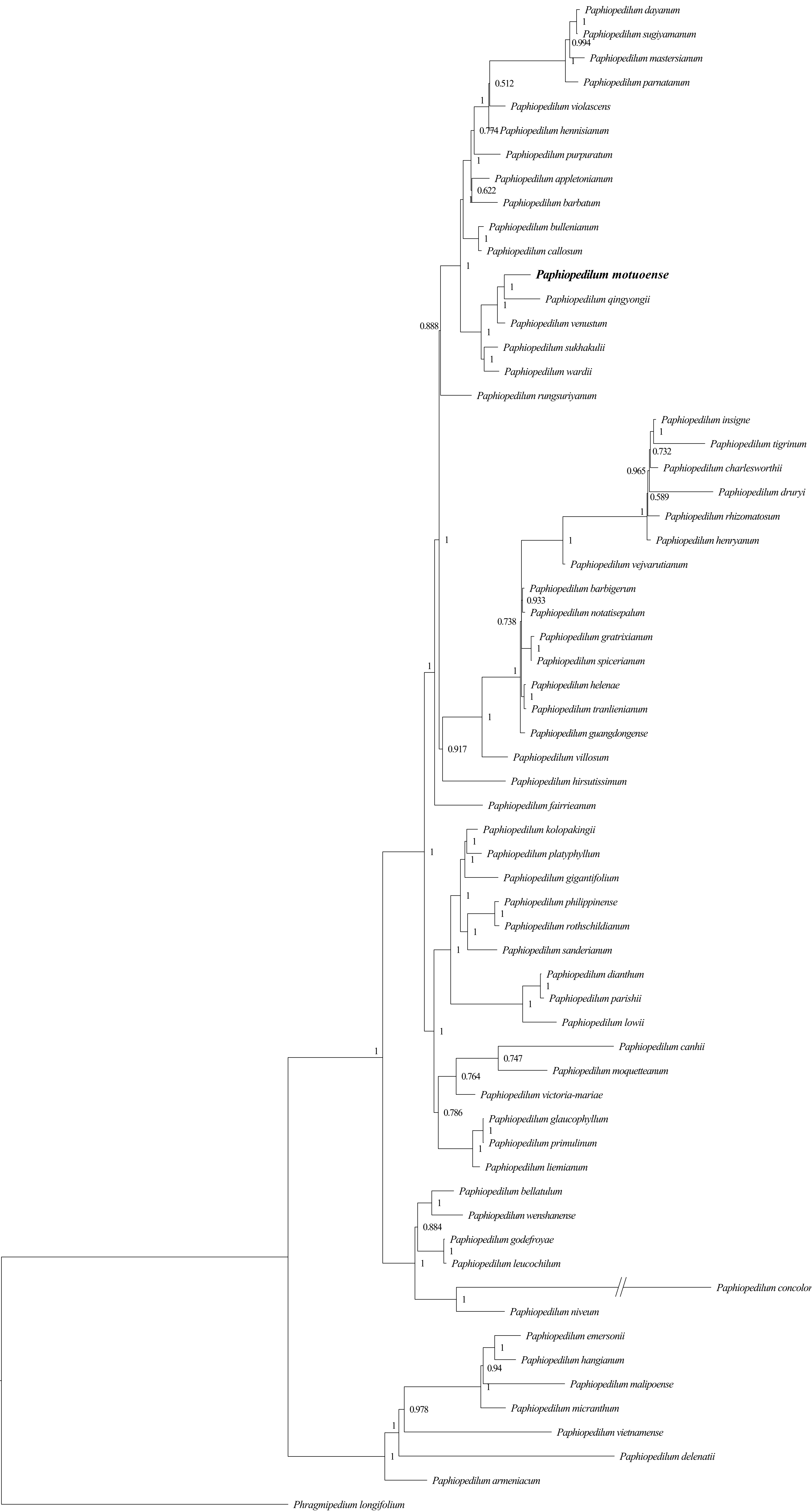

0.003

Supplement: Supplementary material 1 — Bayesian Inference (BI) phylogenetic tree of Paphiopedilum species [file phytokeys-259-131_article-145861__-s001.pdf]
